# Informatics education for translational research teams: An unrealized opportunity to strengthen the national research infrastructure

**DOI:** 10.1017/cts.2022.481

**Published:** 2022-10-28

**Authors:** Eneida A. Mendonca, Rachel L. Richesson, Harry Hochheiser, Dan M. Cooper, Meg N. Bruck, Eta S. Berner

**Affiliations:** 1 Indiana University/Regenstrief Institute, Indianapolis, IN, USA; 2 Cincinnati Children’s Hospital and University of Cincinnati, Cincinnati, OH, USA; 3 University of Michigan Medical School, MI, USA; 4 University of Pittsburgh, Pittsburgh, PA, USA; 5 University of California, Irvine, CA, USA; 6 University of Alabama at Birmingham, Birmingham, AL, USA

**Keywords:** Translational research, education, workforce, informatics, electronic health records, clinical research informatics, needs assessment

## Abstract

**Objective:**

To identify the informatics educational needs of clinical and translational research professionals whose primary focus is not informatics.

**Introduction:**

Informatics and data science skills are essential for the full spectrum of translational research, and an increased understanding of informatics issues on the part of translational researchers can alleviate the demand for informaticians and enable more productive collaborations when informaticians are involved. Identifying the level of interest in different topics among various types of of translational researchers will help set priorities for development and dissemination of informatics education.

**Methods:**

We surveyed clinical and translational science researchers in Clinical and Translational Science Award (CTSA) programs about their educational needs and preferences.

**Results:**

Researchers from 23 out of the 62 CTSA hubs responded to the survey. 67% of respondents across roles and topics expressed interest in learning about informatics topics. There was high interest in all 30 topics included in the survey, with some variation in interest depending on the role of the respondents.

**Discussion:**

Our data support the need to advance training in clinical and biomedical informatics. As the complexity and use of information technology and data science in research studies grows, informaticians will continue to be a limited resource for research collaboration, education, and training. An increased understanding of informatics issues across translational research teams can alleviate this burden and allow for more productive collaborations. To inform a roadmap for informatics education for research professionals, we suggest strategies to use the results of this needs assessment to develop future informatics education.

## Background

From the very inception of NIH’s Clinical and Translational Science Award (CTSA) program and the establishment of the National Center for Advancing Translational Sciences (NCATS), clinical and population health data were viewed as a new frontier of biomedical research, potentially fertile, but underutilized. The vision of transformed research in the new century pictured frontline clinicians stimulated by day-to-day interactions with their patients seeking to fill gaps in biomedical knowledge as rapidly as possible. With data in electronic form, researching the electronic health record (EHR) would be easy for the clinician, with ready access and tools using laptops or workstations. However, the reality of the extensive socio-technical challenges of designing usable systems that could both collect valuable data and integrate data-driven health interventions and innovations in the form of context-relevant clinical decision support quickly emerged. A number of critical challenges including the complexity of accessing EHR data, the lack of interoperable terminologies and data formats, fundamental issues of privacy and data security, and increasingly sophisticated analytical tools have altered the notion of what skills are required for research teams to successfully leverage EHR data and systems for successful clinical and translational research (CTR).

It is now generally accepted that informatics and data science skills are essential for the full spectrum of translational research, supporting hypothesis generation, study planning, data collection, management, analysis, knowledge generation, and knowledge dissemination by integration into practice [1–7]. The foundational role of informatics as infrastructure is further illustrated by the number of informatics faculty and staff who play essential roles at academic medical centers, research networks, and the CTSA consortium and hubs. However, despite the importance of informatics theory and methods, many translational researchers and research staff, especially junior researchers engaged in pilot projects, have little knowledge of the informatics underpinnings of their research. There are many challenges in getting access to data from EHRs, but without knowledge of the way EHR data are obtained, structured, and organized, or knowledge of available tools to facilitate access, the use of these data will continue to be suboptimal.

Despite the presence of informatics experts at each of the CTSA hubs, informatics education for non-informaticians has yet to reach its full potential. While informatics training programs do exist at many sites [8,9], their focus is on training informatics researchers. The leaders of the CTSA training programs, not (typically) being experts in informatics, tend not to prioritize informatics training, leaving an unrealized opportunity to improve clinical research infrastructure.

Although direct collaboration with informatics researchers might seem to be a plausible strategy for closing gaps in translational informatics needs, structural constraints place limits on this approach. Informatics researchers at CTSA institutions are often funded to conduct their own informatics research and may not have extensive involvement in CTSA research and training programs. Furthermore, the active interest in machine learning/artificial intelligence in medicine has led to more demand for informaticians as collaborators. Increased understanding of informatics issues on the part of translational researchers will help move projects further, alleviating the demand for informaticians and allowing for more productive collaborations when informaticians are involved. Just as a working knowledge of statistical techniques can be useful when consulting with statisticians, familiarity with informatics issues will help translational researchers more effectively interact with informaticians.

There is a substantial body of work on the need for greater educational resources for translational researchers [10–14]. Although these efforts often acknowledge the range of stakeholders involved [15], most focus heavily on formal coursework for doctoral or graduate researchers. Several efforts have noted the need for training clinicians in data science, machine learning, and informatics [16–24], often involving hands-on [25], masters’ [26], or fellowship [27,28] experiences. Large-scale community efforts aimed at developing and cataloging educational resources include the recent CTSA DIAMOND portal[29], the CTSA-CLIC Education Clearinghouse [30] and earlier efforts including the OHSU Big data to Knowledge (BD2K) educational materials [31], the BD2K Training Coordination Center [32], and the ONC Health IT Curriculum [33]. However, simply having informatics resources available does not guarantee that they will be used. An assessment of the educational interests and needs of clinical and translational researchers can help inform the reconfiguration of existing materials and the development of new materials to meet those needs. Specifically, identifying the level of interest in different topics among different groups of translational researchers will help set priorities for development and dissemination of educational materials.

## Aim

The aim of our research was to identify the informatics educational needs of CTR professionals whose primary focus is not informatics. This research can inform a roadmap for informatics education for CTR professionals, including a list of critical informatics topics that could be addressed by developing education materials that are accessible and relevant for CTR investigators and staff.

## Methods

### Survey Intent and Design

We conducted a web-based needs assessment survey of CTR researchers and research staff at CTSA hubs to identify informatics and data Science education topics that were of interest to them. For each of these topics, survey respondents were asked to indicate the relevance to their work as well as their perceived need/desire for education on that topic. The complete survey can be found in the Appendix. The survey was developed by the authors, based on their extensive experience in informatics education, and was approved by the Indiana University School of Medicine Institutional Review Board (protocol number 11745). As the goal of the survey was to collect descriptive data, and not to measure generalizable constructs, we did not conduct a formal validation of the instrument [34].

### Sample and Approach

A cover letter to hub leadership was sent to 62 administrators on the CTSA-CLIC administrator listserv asking administrators to distribute the letter that explained the survey to 5–10 researchers at their hub and/or partner network. The letter included the URL for the survey.

### Analyses

Descriptive analyses were used to summarize the educational needs of the respondents and the differences between types/roles of respondents. The data were aggregated across sites and expressed as percentages that reflected the percentage of the total number of respondents for each role who actively indicated interest in a given topic. Those respondents who reported that they were not interested in the topic, or did not know enough about it to answer, or who omitted the item were considered not interested in the topic. All other responses were considered to indicate interest in the topic. The overall percentage is the percentage of total respondents who actively indicated interest in a topic.

## Results

A total of 239 respondents from 23 out of 62 CTSA hubs (37%) responded. Although sites were asked to solicit input from a relatively small number of respondents, some sites solicited more than the minimum number and the number of respondents varied by site and role. Table 1 shows the distribution of roles of respondents and the number of respondents who held each role across all sites.

**Table 1.** Roles of respondents

Respondent role	Number of respondents
Basic Science Researcher	19 (8%)
Clinical Science Researcher	41 (17%)
Outcomes or Policy Researcher	18 (8%)
Translational Science Researcher	40 (17%)
Clinician/Teacher	25 (10%)
Research Staff	48 (20%)
Trainee	30 (13%)
Other	18 (8%)

As Table 1 shows, the respondents include a diverse sample of CTSA stakeholders.

Table 2 shows the interest respondents showed for each of the topics.

**Table 2.** Percentage of respondents reporting interest in topic, overall, and by role

Percentage of respondents reporting interest in learning more about topic										
*Question:*		Overall Percent Interest	Basic Scientists (*n* = 19)	Clinical Scientists (*n* = 41)	Clinicians/Teachers (*n* = 25)	Outcomes/Policy Researchers (*n* = 18)	Research Staff (*n* = 48)	Translational Scientists (*n* = 40)	Trainees (*n* = 30)	Other (*n* = 18)
Q3	Understanding data									
Q3-1	Clinical data (e.g., medications, labs)	71.5%	63.2%	73.2%	64.0%	88.9%	72.9%	62.5%	80.0%	72.2%
Q3-2	Genetics/genomic data	67.4%	68.4%	70.7%	56.0%	44.4%	66.7%	80.0%	70.0%	66.7%
Q3-3	Public/population health data	75.7%	63.2%	78.0%	60.0%	94.4%	75.0%	72.5%	90.0%	72.2%
Q4	Collecting data									
Q4-1	Case report forms, questionnaires, other data collection tools	68.2%	52.6%	68.3%	64.0%	83.3%	77.1%	52.5%	76.7%	72.2%
Q4-2	Recruitment and retention	74.9%	57.9%	80.5%	56.0%	94.4%	79.2%	72.5%	80.0%	72.2%
Q5	Managing data									
Q5-1	Data storage and exchange	71.5%	78.9%	65.9%	56.0%	66.7%	68.8%	85.0%	76.7%	72.2%
Q5-2	Assessing and reporting data quality	74.9%	68.4%	75.6%	52.0%	88.9%	77.1%	75.0%	86.7%	72.2%
Q5-3	Integrating EHR data w/other data types	73.2%	47.4%	75.6%	64.0%	83.3%	79.2%	77.5%	73.3%	72.2%
Q5-4	Techniques for mining large data sets	72.4%	78.9%	70.7%	60.0%	44.4%	72.9%	90.0%	76.7%	66.7%
Q5-5	Managing qualitative data	66.5%	52.6%	58.5%	64.0%	77.8%	75.0%	60.0%	76.7%	66.7%
Q6	Analyzing data									
Q6-1	Identifying patient cohorts w/EHR data	67.4%	47.4%	68.3%	64.0%	66.7%	66.7%	72.5%	76.7%	66.7%
Q6-2	Analysis of genomic and biological data	62.8%	73.7%	68.3%	52.0%	33.3%	62.5%	72.5%	63.3%	61.1%
Q6-3	Analysis of clinical data	68.6%	57.9%	61.0%	64.0%	77.8%	68.8%	75.0%	76.7%	66.7%
Q6-4	Rigor and reproducibility in data analysis	73.6%	73.7%	73.2%	64.0%	72.2%	64.6%	90.0%	80.0%	66.7%
Q6-5	Identifying and managing adverse events	60.3%	42.1%	53.7%	60.0%	50.0%	66.7%	67.5%	66.7%	61.1%
Q6-6	Data mining and machine learning	68.6%	68.4%	75.6%	64.0%	72.2%	60.4%	80.0%	60.0%	66.7%
Q6-7	Text mining and natural language processing	59.8%	42.1%	68.3%	60.0%	61.1%	56.3%	65.0%	56.7%	61.1%
Q6-8	Data visualization approaches	72.8%	68.4%	75.6%	60.0%	83.3%	56.3%	92.5%	76.7%	72.2%
Q6-9	Public health data analytics	65.7%	42.1%	70.7%	56.0%	72.2%	62.5%	75.0%	66.7%	72.2%
Q6-10	Analyzing qualitative data	62.3%	52.6%	61.0%	56.0%	72.2%	66.7%	62.5%	63.3%	61.1%
Q7	Applying/managing knowledge									
Q7-1	Computable and Clinical Decision Support (CDS) knowledge	54.0%	52.6%	53.7%	68.0%	38.9%	52.1%	62.5%	50.0%	44.4%
Q7-2	Integrating guidelines or CDS into Electronic Health Record	58.2%	26.3%	65.9%	60.0%	61.1%	60.4%	60.0%	56.7%	61.1%
Q8	Managing research projects									
Q8-1	Project management	70.7%	57.9%	68.3%	76.0%	72.2%	75.0%	67.5%	76.7%	66.7%
Q8-2	Planning, management and leadership for health information technology	56.9%	42.1%	58.5%	72.0%	44.4%	66.7%	45.0%	53.3%	66.7%
Q8-3	Working in diverse teams	69.0%	73.7%	61.0%	68.0%	61.1%	75.0%	72.5%	73.3%	61.1%
Q9	Reporting and sharing data									
Q9-1	Trial registries and results reporting	61.5%	47.4%	58.5%	64.0%	66.7%	64.6%	60.0%	70.0%	55.6%
Q9-2	Policies and platforms for data sharing	69.9%	52.6%	61.0%	68.0%	83.3%	72.9%	75.0%	76.7%	66.7%
Q9-3	Publication and presentation	63.6%	68.4%	48.8%	68.0%	55.6%	72.9%	65.0%	70.0%	55.6%

Responses indicate a strong interest in the 30 data science and informatics topics included in the survey. The topics with highest overall percentage of interested respondents were as follows: Understanding Public/Population Health Data (75.7%), Collecting Data for Recruitment and Retention (74.9%), and Assessing/Reporting Data Quality (74.9%). The topics with the lowest overall interest were Computable/Clinical Decision Support (CDS) Knowledge (54%), Planning, Management and Leadership for Health IT (56.9%), and Integrating Guidelines/CDS into the EHR (58.2%). For almost all topics, the majority of respondents from most roles reported some interest in the topic. One exception is the topic of Computable and CDS Knowledge, which was of less interest (ranging from 38.9% to 52.6% by role) to basic scientists and clinical scientists, outcomes/policy researchers, research staff, trainees, and others, although clinician teachers reported higher interest (68%). Basic scientist respondents showed lower interest (<57%) in the greatest number of topics (15) than respondents in other roles. Clinicians/teachers also reported lower interest (<57%) in seven different topics. Outcomes/policy researcher respondents and trainees reported very high interest (>80%) in the largest number of topics, with outcomes/policy researchers reporting very high interest (>80%) in eight different topics, high interest (57–79%) in 15 topics, and low interest (<56%) in 7 of the 30 topics on the survey. Trainees reported very high interest in 4 topics, high interest in 22 topics, and low interest in 4 topics.

There were several topics where organizational role appears to be a predictor of interest. For the topic of “Managing Genetics/Genomic Data,” Outcomes/policy researchers (44.4%) and clinicians/teachers (56%) reported less interest compared to other research roles, which ranged from 66.7 to 80% of respondents interested in the topic. Similarly, respondents reporting an outcomes/policy role reported less interest in training in “Analysis of Genomic and Biological data” (33.3%) where the interest of the other respondents in that topic ranged from 52% to 73.7%.

## Discussion

Although many CTSA hubs have informatics offerings for non-informatician researchers, the needs and interests of non-informatics researchers with respect to informatics topics have not been well studied. Our data suggest that training and education in data science and clinical informatics for translational scientists have failed to keep pace with the universal implementation of the EHR and advances in computing that have taken place over the past decades. Lawrence Weed in his prescient 1968 articles that transformed the structure of physician notes in the medical chart heralded the onset of a new age of data collection and management empowered by advances in computer technology [35,36]. Weed noted, “Among physicians there has been uncritical adherence to tradition in the first phase of medical action, which is the collection of data, upon which complete formulation and management of all the patient’s problems depends” [35]. Weed continued, “A more realistic goal in teaching [clinicians] is to discipline the physician in the most effective application and growth of his own developing store of factual information through his own disciplined study of actual cases. The computer can make an enormous contribution in this area” [36]. This is certainly true for researchers in general and in particularly, translational science researchers whose research interests span a broad range of the translational science spectrum.

Our survey of participants from CTSA hubs showed that a large number of researchers were interested in learning more about a variety of informatics topics. On average 67% of respondents across roles and topics expressed interest in learning more, and even the topic with the least interest had over half of respondents expressing interest. Respondents in some roles expressed less interest in certain topics, and often these topics and roles were at opposite ends of the translational science spectrum. For instance, outcomes researchers were less interested in the bioinformatics topics related to genomics, while these topics were more clearly of interest to basic science researchers. Respondents in the different roles varied in the topics identified as being of interest, suggesting that providing education on a broad array of topics would likely address the needs of a large number of individuals, but any one topic or set of topics is likely to interest only a few.

The topic of Computable Knowledge and Clinical Decision Support received the lowest interest which is surprising given its growing importance. In looking at the survey, we realized that we had abbreviated Clinical Decision Support as CDS, and it is very possible that respondents did not recognize the abbreviation. This may mean that we may not have accurate data on interest in this topic, which is a limitation. On the other hand, this topic may be more of a clinical topic and of less interest to researchers, since 68% of clinical teachers did report an interest in it.

The need for better-integrated and focused training in informatics is critical to optimize communication among the many disciplines that constitute the health care and research ecosystem. Lessons learned from team science research strongly suggest that a common lexicon, the essential “terminology harmonization,” is in and of itself an essential component of successful multidisciplinary endeavors [37]. The need for more wide-reaching informatics training extends beyond an initial familiarization across the range of the health care workforce (nurses, physicians, researchers, medical therapists, etc.). Innovations in technology have inevitably led to new career pathways (e.g., the discovery of the imaging power of X-rays and the field of radiology), and we are witnessing a similar evolution in the health care and research workforce as data science knowledge and practice expands. For example, clinical informatics is a formally recognized medical subspecialty with a highly structured set of patient-oriented and didactic requirements [38–41]. Career opportunities in nursing informatics are also growing rapidly (e.g., https://nurse.org/resources/nursing-informatics/). From a translational research perspective, it is clear that strategies are underway to increase the biomedical informatics workforce with particular attention on expanding workforce diversity [42]. However, the majority of translational researchers are not, and will not be, informatics experts, but they will need to work collaboratively with these experts. The intent of the education envisioned in this needs assessment survey would not be to make these researchers into formally trained informaticians, but to provide them with the knowledge and skills to promote effective collaboration with informaticians and, in some cases, perform tasks that previously might have required assistance from informatics experts.

What, then, might our survey inform in terms of next steps in informatics training for translational researchers? Clearly, a single training module or paradigm is unlikely to meet the variety of specific informatics learning goals of the diverse research workforce. Novel approaches to integrate informatics training into the ecosystem are emerging. For example, Chen et al. published a medical student led elective in the growing field of clinical informatics that focused on experiential learning, the kind of “hands-on” training that is embedded in medical student training [43]. Addressing the increasing stresses associated with medical subspecialty training, Saville et al. proposed a flexible approach to additional training that included clinical informatics [44]. By carefully integrating the additional learning modules in the busy schedule demanded in medical subspecialty training, the authors found that the program succeeded in preventing stress and burnout as well as propelling individuals toward lifelong and rewarding careers.

There are some publicly available resources [29–31,33] that include a number of informatics and data science topics that could be repurposed to more closely target the need for informatics education for non-informatics researchers, but these sources do not cover all of the topics of interest. Informatics researchers at the different CTSA hubs could probably address many of these topics, but their time for education of the broader research community is limited. An effort across CTSA hubs leveraging the informatics expertise at each hub, but not overburdening any one hub, may be needed. Self-instructional materials that could be used across hubs is another alternative. Existing repositories could be repurposed to focus on the unique informatics needs of translational researchers, and new materials might need to be developed and be made publicly available. Our results provide data on potential foci for educational materials for different translational science researcher roles.

In recent years, the concept of the “learning health system” (LHS) has emerged as a novel collaborative framework in which to address unsolved problems in health and medicine [45]. The LHS paradigm is quintessentially team science in that it requires the active involvement of content experts, community stakeholders, patients, and policy-makers all embedded in what has been called a “virtuous cycle” of state-of-the-art data acquisition, dissemination, and implementation. Our survey revealed that a diverse pool of potential learners expressed interest in training across a wide range of informatics-related topics that included rigor and reproducibility in data analyses, working in diverse teams, and genetics and genomic data. Thus, we would suggest that simply providing a list of courses and training opportunities in individual topics, while essential, may not empower a more effective and integrated approach to informatics learning. Kohn et al. recently suggested that the approach to informatics training constitutes in and of itself a LHS [6]. They posited three distinct elements of the institutional biomedical informatics LHS:

- Catalyze: Integrate resources for research informatics across the health center, and synergistically support the informatics needs of other affiliated and community-based research centers, departments, and institutes.
- Discover: Facilitate biomedical discovery across the spectrum of informatics, including bioinformatics, imaging, clinical, translational, and public health informatics, and develop a portfolio of externally funded research in the area of biomedical informatics.
- Educate: Support and inspire the training of the next generation of investigators in the principles and practice of biomedical informatics.

In summary, our data support thoughtful and strategic deployment of institutional resources to advance training in the broad areas of clinical and biomedical informatics. We believe that the success of such programs ultimately will be tied to a broader, institutional commitment to embedding data science and clinical informatics in all aspects of health, clinical care, and research.

## Recommendations

As the complexity and use of information technology and data science in research studies grows, informaticians will continue to be a limited resource for research collaboration, education, and training. An increased understanding of informatics issues across the research team can alleviate this burden and allow for more productive collaborations.

Given our findings and the growing importance of cross-trained multidisciplinary research teams, we suggest a number of recommendations for CTSAs and other research organizations to advance informatics and data science education and training programs for clinical researchers and staff. These include:

1) **Recognize, articulate, and promote the value of informatics training for all members of clinical and translational science teams**. Training and services that support researchers and staff develop and conduct research are a vital infrastructure component for academic medical centers and organizations striving to be LHSs. Increased understanding of informatics issues across the clinical and translational science team can improve the speed and efficiency of research proposals and projects and enable more productive collaborations with informaticians. CTSA sites can be more “welcoming” and accessible to non-informatics research staff by articulating the importance of professional development in this area and introducing a range of topics in non-informatics jargon. Additionally, leaders of research support services might identify local individuals in clinical or research roles who have mastered and applied informatics skills to advance their research. The proposed training should be adapted to the targeted audience. For example, to engage busy frontline clinicians, training modalities might include brief introductions at clinical grand rounds (for which attendance by clinicians is typically robust, if not mandated). Brief webinars that offer CME credits may, for some clinicians, be appealing as well. Finally, the informatics community could encourage the development of formal fellowship training in clinical informatics for those Academic Health Centers (AHC) with the inclination and resources to support these types of programs [46].

2) **Review and encourage the use of content and educational materials** that might apply across the range of clinical and translational science. The categories and topics of our survey provide a good starting point for understanding, organizing, and offering/delivering the full range of informatics training and skills. These topics can support local and national needs assessments and inform a roadmap to develop informatics training materials that are useful to a wide range of non-informatics researchers and staff. The informatics faculty at a particular AHC might organize a “community engagement studio” for interested parties to articulate the topics of keen interest and help guide the development of outreach efforts [47].

3) **Join and support national efforts, through the CTSA consortium, to develop shared offerings** that can be implemented to address local needs. We have identified some topics for which publicly available training resources do exist [29–33], but existing training resources need to be organized and presented into a coherent framework that is relevant to the questions and work of clinicians and clinical researchers so that learners can easily identify what they need, and what is available. For topics that do not yet have good basic educational materials, a national consortium could coordinate efforts to ensure that educational materials can be developed to address each area. A coordinated roadmap for development is needed, as well as incentives to share training and to coordinate the development of research-relevant collections of informatics education and training modules and activities.

4) **Dedicate effort to customize and integrate these educational offerings into actual research contexts and activities to increase local adoption and impact**. Researchers are busy and education is more valuable when it addresses an immediate and applied need. Local CTSA sites can minimize the resources/time invested in developing and offering skills training by using shared resources. Organizations can embed informatics training resources in existing information about local CTSA resources. An additional incentive might be to encourage CTSA pilot studies that include collaboration between clinicians and informaticians.

5) To adapt to changes in the field**, each site should regularly monitor researcher and staff needs for education, training, and informatics support services**. The CTSA consortium should develop mechanisms to share this feedback across sites and bring it to the national CTSA consortium level to address. One might consider the creation of a national “learning health system” focused on continuous quality improvement of clinical and biomedical informatics training.

6) **Routinely evaluate informatics education offerings and use this information to continuously improve educational materials** and adapt the curricula to changing needs and emerging topics.

7) **Explore incentives for researchers and staff** to partake in informatics training and reduce barriers to requesting and accessing needed training. Similarly, explore incentives for informaticians to develop and share education and training modules related to improving CTR. The use of formal certification and micro-badges in select topics might prove useful in this context [48].

## Limitations

There are some limitations to this study. Although the respondents represented a variety of roles across many of the CTSA sites, our respondents only represented 37% of the sites and we do not know how many individuals were actually solicited. However, it is likely that those who responded were the people most interested in informatics education, and their responses indicate that there is a need for informatics education beyond those specializing in informatics. While our response rate might appear to be low, this is actually reasonable and expected compared with other email surveys. A review of recent literature on generalizability theory and survey response rates across a variety of the medical literature suggests that a response of about 40% is considered to be acceptable. Moreover, there is also recent literature suggesting that extensive, costly, and time-consuming efforts to increase response rates above acceptable magnitudes do little to improve the accuracy or generalizability of the survey [34,49–51]. Also, although our survey instrument did not go through a formal validation process, we believe that our survey captures the necessary breadth of informatics topics, as it was informed by our collective experience as informatics researchers and educators, as well the content of our published textbooks in informatics [52,53]. We also did not explicitly ask respondents what services they would like their informatics team to provide to them nor did we investigate the offerings or requests at individual sites. It is possible that services are provided at a site, but respondents are interested in more education for themselves on the given topics. We presume that a reported interest in a topic implies a gap in educational or training options at the site, but further research would be necessary to test this assumption. We also have not conducted a systematic assessment of currently available educational resources, how those resources are used, and how those usage patterns might relate to patterns of informatics referrals at the hub. Further assessment of these issues would likely lead to increased nuance in our understanding of educational needs and should be considered in future work.

## Conclusions

Informatics education for clinical and translational scientists and research teams is vital to support efficient research that can leverage EHR data and systems. Some resources already exist and provide a starting point for educating the clinical and translational science workforce. Our survey appears to be the first to assess the topic of informatics training for non-informatics researchers, and our analysis has revealed gaps and a starting set of recommendations to better understand and address these gaps to optimize the CTR and evidence generation process.

The identification of researcher needs described here can be used to further refine existing educational resources and to provide a roadmap for the development of new informatics education materials targeted specifically to translational science researchers. Research sponsors and training programs, including CTSA and others, can enable the development of informatics education and training and ensure that it addresses and evolves with researcher needs, ultimately providing badly needed infrastructure to improve the speed and efficiency of CTR across the nation.

## Supplementary material

For supplementary material accompanying this paper visit https://doi.org/10.1017/cts.2022.481. click here to view supplementary material
