# Supplementary material for: Informatics education for translational research teams: An unrealized opportunity to strengthen the national research infrastructure
Source: J Clin Transl Sci. 2022 Oct 28;6(1):e130. doi: 10.1017/cts.2022.481 (PMC9794970; doi:10.1017/cts.2022.481)
Supplement: Supplementary file 1 [file S2059866122004812sup001.pdf]

APPENDIX

INFORMATICS EDUCATION NEEDS ASSESSMENT SURVEY

We're trying to understand the challenges that translational scientists have with the collection, representation, formatting, and coding" of various commonly-found data types. Please let us know about your level of interest and knowledge of these types of data."

What is the institution you are affiliated with? _______________________________________

What is your primary role at the institution?

Basic science researcher
Translational science researcher
Clinical science researcher
Outcomes or policy researcher
Basic science educator
Clinician/teacher
Trainee (student/post-doc in the health sciences)
Research staff
Other (specify)________________

		I want to learn what this topic is about	I know what the topic is about and I want to find out how it applies to my work	I am already using the topic in my work and want more depth of knowledge in it.	The topic is a focal area of my own work and I want more advanced educational materials.	Not interested in this topic or don't need additional training	I have not heard of this topic and cannot answer.
**Q3**	**Understanding Data**						
Q3-1	Clinical data (e.g., medications, labs)						
Q3-2	Genetics/genomic data						
Q3-3	Public/population health data						
**Q4**	**Collecting Data**						
Q4-1	Case Report Forms, questionnaires, and other data collection tools						

Q4-2	Participant Recruitment & Retention - Informatics Approaches and Tools -reaching diverse and understudied populations						
**Q5**	**Managing Data**						
Q5-1	Fundamentals of data storage and exchange						
Q5-2	Assessing and reporting data quality						
Q5-3	Integrating EHR data with other types of data						
Q5-4	Techniques for managing large data sets e.g., genetic/genomic, imaging data						
Q5-5	Managing qualitative data						
**Q6**	**Analyzing Data**						

Q6-1	Identifying patient cohorts using EHR data						
Q6-2	Analysis of genomic and biological data						
Q6-3	Analysis of clinical data						
Q6-4	Assuring rigor and reproducibility in data analysis, including identification and reduction of bias						
Q6-5	Identifying and managing adverse events						
Q6-6	Data mining and machine learning						
Q6-7	Text mining and Natural Language Processing						
Q6-8	Data visualization approaches						
Q6-9	Public Health data analytics						

Q6-10	Analyzing qualitative data						
**<u>Q7</u>**	**Applying/Managing Knowledge**						
Q7-1	Fundamentals of computable biomedical knowledge and CDS knowledge						
Q7-2	Integrating clinical guidelines or clinical decision support into EHR						
**<u>Q8</u>**	**<u>Managing</u> Research Projects**						
Q8-1	Project Management						
Q8-2	Planning, Management and Leadership for Health IT						
Q8-3	Working in Diverse, Interdisciplinary and Inter-institutional Teams						

Q8-4	Other topics not mentioned above (specify)						
**Q9**	**Reporting and Sharing Data**						
Q9-1	Registries of Clinical Trials and trial results reporting						
Q9-2	Policies and platforms for data sharing						
Q9-3	Publication and presentation						
**Q10**	**Other Topics**						
Q10-1	Other (specify)						
